# Association Between PM_2.5_ and Daily Hospital Admissions for Heart Failure: A Time-Series Analysis in Beijing

**DOI:** 10.3390/ijerph15102217

**Published:** 2018-10-11

**Authors:** Man Li, Yao Wu, Yao-Hua Tian, Ya-Ying Cao, Jing Song, Zhe Huang, Xiao-Wen Wang, Yong-Hua Hu

**Affiliations:** 1 School of Public Health, Peking University, Beijing 100191, China; liman1993@bjmu.edu.cn (M.L.); yaowu@pku.edu.cn (Y.W.); yaohua_tian@bjmu.edu.cn (Y.-H.T.); cao90906130@pku.edu.cn (Y.-Y.C.); jingsong91@bjmu.edu.cn (J.S.); huangzhe@bjmu.edu.cn (Z.H.); wangxw@bjmu.edu.cn (X.-W.W.); 2 Peking University Medical Informatics Center, Beijing 100191, China

**Keywords:** air pollution, PM_2.5_, heart failure, short-term association

## Abstract

There is little evidence that acute exposure to fine particulate matter (PM_2.5_) impacts the rate of hospitalization for congestive heart failure (CHF) in developing countries. The primary purpose of the present retrospective study was to evaluate the short-term association between ambient PM_2.5_ and hospitalization for CHF in Beijing, China. A total of 15,256 hospital admissions for CHF from January 2010 to June 2012 were identified from Beijing Medical Claim Data for Employees and a time-series design with generalized additive Poisson model was used to assess the obtained data. We found a clear significant exposure response association between PM_2.5_ and the number of hospitalizations for CHF. Increasing PM_2.5_ daily concentrations by 10 μg/m^3^ caused a 0.35% (95% CI, 0.06–0.64%) increase in the number of CHF admissions on the same day. We also found that female and older patients were more susceptible to PM_2.5_. These associations remained significant in sensitivity analyses involving changing the degrees of freedom of calendar time, temperature, and relative humidity. PM_2.5_ was associated with significantly increased risk of hospitalization for CHF in this citywide study. These findings may contribute to the limited scientific evidence about the acute impacts of PM_2.5_ on CHF in China.

## 1. Introduction

Congestive heart failure (CHF) is a serious public concern because of its poor prognosis [1] and considerable economic impact on health services, given that it affected 40.0 million people worldwide in 2015 [2]. Epidemiological studies from developed countries have extensively reported that acute exposure to fine particulate matter (PM_2.5_) has a close temporal association with hospitalization for and mortality from CHF [3,4,5], especially in patients with histories of diabetes [6] and hypertension [7]. A meta-analysis published in 2013 reported that increasing PM_2.5_ daily concentrations by a unit of 10 μg/m^3^ would contribute to an estimated 2.12% increase in hospitalization for CHF and CHF mortality globally [5]. Thus, effective control of PM_2.5_ would yield significant public health benefits. In the USA, reduction of average concentrations of daily PM_2.5_ by 3.9 μg/m^3^ would reportedly decrease the number of hospitalizations for CHF by almost 8000 and save around 300 million dollars in related healthcare costs annually [5].

Air pollution is known to be more severe in developing and low- and middle-income countries, and geographical and temporal differences are known to exist in the effects of PM_2.5_ [8]. Therefore, the findings from developed countries such as the USA and European countries cannot be directly generalized to all developing countries. There is little evidence concerning the effects of acute exposure to PM_2.5_ on hospitalization for CHF in developing countries because there is poor environmental monitoring and disease surveillance data in these countries [5,9]. The meta-analysis mentioned above that addresses the relationship between PM_2.5_ and hospitalization for CHF includes only one relevant study from developing countries [5]. To the best of our knowledge, only two studies thus far have investigated the association between PM_2.5_ and hospitalization for CHF in China [10,11]. Moreover, the effects of PM_2.5_ on cardiovascular risk in the setting of severe air pollution is still unclear [12]. Thus, the aim of this retrospective study was to assess short-term associations between PM_2.5_ and hospitalization for CHF in Beijing from 2010 to 2012 by using a time-series design. Our findings may have implications for relevant policy formulation, clinical decision making, and prevention of and interventions for CHF.

## 2. Materials and Methods

### 2.1. Data on Hospitalization for CHF

We obtained data on CHF hospitalization from Beijing Medical Claim Data for Employees, which covers all working and retired employees. Sex, date of birth, dates of hospital visits, medication use, discharge diagnoses in Chinese, and corresponding International Classification of Diseases 10th version (ICD-10) codes are all contained in this database, the details of which have been described previously [13]. In the present study, the primary study cohort included only adults. We extracted daily hospital admissions with a primary diagnosis of CHF (ICD-10 code I50) between 1 January 2010 and 30 June 2012 (a total of 912 days) from the database. The selection of the study period was on the basis of availability of both air pollution and health data. Individuals’ detailed information on the disease diagnosis were required to identify the CHF admission. Days without information on ICD code for CHF could not be classified as hospitalizations for CHF. The present study did not require Institutional Review Board approval or participant consent because the data used were collected for administrative purposes and included no personal identifiers.

### 2.2. Environmental Data

We collected relevant daily PM_2.5 _ data from the air pollution reports issued by the US Embassy (https://china.usembassy-china.org.cn/zh/), which established an air quality monitoring station on the rooftop of the embassy building located in Chaoyang District, Beijing. Daily meteorological data on temperature (°C) and relative humidity (%) from the Chinese Meteorological Bureau (http://data.cma.cn/) were also collected. A previous study [14] reported that PM_2.5_ levels obtained from the US embassy’s monitor were roughly comparable with city-wide PM_2.5_ levels. In addition, other studies [15,16] reported that the maximum distance from the monitor to hospitals can be considered about 40 km for the purpose of minimizing misclassification of exposure. According to Xie et al. [12], in all high-density population areas (>5000 people/km^2^), 97.8% (44/45) of the tertiary hospitals and 79.3% (69/87) of the secondary hospitals in Beijing are located within a 40-km radius of the monitor. Additionally, the application and reliability of the US embassy’s monitoring point data has been discussed in previous studies [13,16]. Until 2013, China has gradually introduced PM_2.5_ in the national air quality monitoring network and publicized real-time monitoring data. Therefore, data from the US embassy was the only publicly available source for daily PM_2.5_ measurements during the study period. It is important to note that daily (24-h) mean concentrations of PM_2.5_ were intended as a proxy for population exposure levels in the current study.

### 2.3. Statistical Analysis

With PM_2.5_ concentrations, meteorological data, and CHF hospitalizations linked by date, we performed a time-series analysis in combination with a generalized additive Poisson model to assess the short-term association between PM_2.5_ and CHF hospitalization in the present study. Confounding covariates incorporated in the model included temperature, relative humidity, public holiday, and day of the week, which is predefined according to previous published studies. We used the following formula in this study:

Log [E(Yt)] = α + βPM_2.5_ + public holiday + day of week + ps(calendar time, 7 per year) + ps(temperature, 3) + ps(relative humidity, 3). (1)

Here t denotes the day of the observation, E(Yt) refers to the expected daily case counts of hospitalization for CHF on day t; ps () denotes the smoother based on the penalized spline function; public holiday is categorized as a dummy variables (0 indicates no holiday, and 1 indicates a holiday), and the day of week on day t is adjusted as a categorical variable; β represents log-relative risk of CHF morbidity in relation to unit increase in PM_2.5_ concentrations;  α is the intercept term.

We controlled for seasonality and time trends using a penalized spline with 7 degrees of freedom (df) per year to exclude unmeasured time trends longer than 2 months in hospital admissions for heart failure. The selection of 7 df per year for calendar time was based on the parameter used in several recent large national studies in China [17,18,19]. We adjusted for the non-linear and delayed effects of weather conditions on admissions for CHF by fitting penalized splines with 3 df for the 3-day moving average air temperature and relative humidity. We also incorporated indicator variables for public holidays and day of the week to adjust for the difference in the baseline hospital admission rates for each day. We have also conducted stratified analyses to explore the potential confounding effects of age and sex. To test the robustness of results, we also conducted sensitivity analyses in terms of df values for time trend (4–8 per year), temperature (2–6), and relative humidity (2–6) [13].

In line with previous studies, we explored non-linear exposure-response association using a penalized cubic regression spline of PM_2.5_ concentration with three degrees of freedom, because of the assumption that linear association between PM_2.5_ level and number of hospitalizations may be not justified [13]. We also assessed the temporal association between PM_2.5_ and hospitalization for CHF by setting up models with a single-day lag from the current day (lag 0) up to previous 3 days (lag1, lag2, and lag3), and with 2-day (lag 0–1), 3-day (lag 0–2), and 4-day (lag 0–3) moving average concentrations. Subgroup analyses were conducted stratified by age group (18–64 years and ≥65 years) and sex. 

The characteristics were presented with as mean ± standard deviation (SD) for continuous variables and percentages (%) for categorical variables. The estimated effects were expressed as the percentage changes and 95% confidence intervals (CIs) in daily CHF visits associated with 10 μg/m^3^ increase in the daily PM_2.5_ concentration. All analyses were conducted using the R Programming Language (Version 3.2.2, R Foundation for Statistical Computing, Vienna, Austria) with the “mgcv” and “nlme” package.

## 3. Results

Table 1 summarizes the descriptive characteristics of patient hospitalization for CHF. From 1 January 2010 to 30 June 2012, we identified 15,256 hospitalizations for CHF, 79.0% of which were of patients aged ≥65 years and 55.9% were male patients.

Table 2 presents a summary of the distribution of hospitalizations for CHF, daily PM_2.5_ concentrations, and meteorological conditions. During the study period, there were 16.7 ± 12.1 hospitalizations per day and the average daily concentration of PM_2.5_ was 99.5 ± 75.3 μg/m^3^ (ranging from 7.2 μg/m^3^ to 492.8 μg/m^3^). The target in the WHO Air Quality Guidelines (24-h average concentration ≤ 25 μg/m^3^) was achieved in 124 days (13.6%).

**Figure 1:**
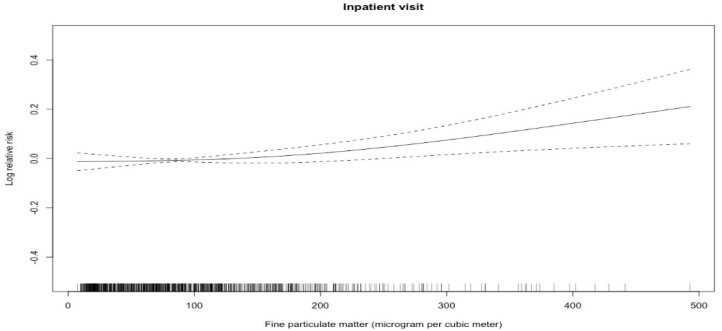
Concentration response curves for PM_2.5_ and hospitalization for CHF. Note: Concurrent day PM_2.5_ concentrations (μg/m^3^) are on the X-axis, and predicted log (relative risk) (RR) on the Y-axis. The concentration response curve is the solid line and the 95% CI are presented by the dotted lines.

Figure 1 shows short-term exposure-response associations between PM_2.5_ and hospitalization for CHF. We noted a broadly linear association between PM_2.5_ and hospital admissions for CHF though the association was relatively flat at PM_2.5_ <100 mg/m^3^, whereas Table 3 summarizes the regression results of single-pollutant models for CHF hospitalization after controlling for some confounders (temperature, relative humidity, public holiday and day of the week). We found significant temporal associations between PM_2.5_ and hospitalization for CHF except for on lag three days. Specially, a 10 μg/m^3^ increase in PM_2.5_ concentration on the same day corresponded to 0.35% (95% CI, 0.06–0.64%) increase in the number of hospitalizations for CHF.

Table 4 shows the short-term sex- and age-specific effects of PM_2.5_ on admission for CHF. The associations between PM_2.5_ and admission for CHF were marginally significant for women (0.43%, 95% CI, −0.005–0.87%) and individuals aged ≥65 years (0.32%, 95% CI, −0.005–0.65%). Table 5 presents the results of sensitivity analyses by changing the df of calendar time, temperature, and relative humidity. We obtained similar regression results after controlling for confounding covariates, which indicated that the association of PM_2.5_ with CHF hospitalization was robust.

## 4. Discussion

In this retrospective study, we examined short-term associations between PM_2.5_ with hospital admissions for CHF between January 2010 and June 2012 in Beijing, China. In the study we noted a broadly linear association between PM_2.5_ and hospital admissions for CHF though the association was relatively flat at PM_2.5_ <100 mg/m^3^. Similarly, a time-stratified case-crossover study for 26 large cities in China also reported the similar trend in association of short-term exposure to PM_2.5_ and CHF hospitalizations [11]. This is also consistent with the curves for the associations between PM_2.5_ and asthma and ischemic stroke observed in our previous studies [15,16]. We found a statistically significant association between acute exposure to PM_2.5_ and hospitalizations for CHF with clear exposure response. We estimated that increasing PM_2.5_ daily concentrations by 10 μg/m^3^ would cause a 0.35% (95% CI, 0.06–0.64%) increase in CHF admissions on the same day. Women patients and individuals aged ≥65 years were more vulnerable to PM_2.5_. These associations remained significant in sensitivity analyses involving changing the degrees of freedom of calendar time, temperature, and relative humidity.

It is well-documented that exposure to air pollution is significantly positively associated with hospital admissions for CHF in developed countries [5,20,21]. For example, Jennifer et al. [21] found a 1-ppm increase in CO was associated with a 3.6% increase in ischemic heart disease admission in persons with a secondary diagnosis of CHF on the same day. However, few studies have specially investigated the short-term effects of PM_2.5_ on hospitalization for CHF [3,5,22,23]. For example, Belleudi et al. [22] found an immediate impact (at lag 0) of PM_2.5_ on hospitalization for CHF (2.4%, 95% CI, 0.3–4.5%) in Rome. Similarly, another study in an Australian state-wide setting reported that PM_2.5_ concentration was detrimentally associated with the incidence of CHF (RR = 1.29, 95% CI, 1.15, 1.42) and of readmission (RR = 1.07, 95% CI, 1.02–1.17 [3]. A study based on a national database from the United States Medicare files further found that CHF had a stronger association with PM_2.5_ than other cardiovascular and respiratory diseases [24]. A recent meta-analysis also demonstrated that increasing PM_2.5_ daily concentrations increased the risk of hospitalization for CHF and mortality [5]. However, one study failed to identify a significant association between PM_2.5_ and hospitalization for CHF, finding that PM_2.5_ increases tended to be associated with fewer hospitalizations; this tendency not being statistically significant [25]. The discrepancy might be attributable to different outcomes definitions (individual-based case-crossover studies and those identified from larger admissions databases), different sample sizes, and higher heterogeneity of CHF.

Because there are geographical and temporal differences in the effects of PM_2.5_ on health outcomes [8], it is meaningful to specifically study the effect of PM_2.5_ on hospitalization for CHF in China. Yang et al. [26] provided the first population-based epidemiologic evidence in China that PM_10_, SO_2_, and NO_2_ may be important risk factors of exacerbation of CHF. Hsieh et al. [10] estimated that an increase in CHF hospitalizations is associated with an interquartile range (IQR) increase in PM_2.5_ of 13% (95% CI, 9–17%) on warm days and 3% (95% CI, 0–7%) on cool days in Taipei, Taiwan. Recently, a time-stratified case-crossover study for 26 large cities in China with 105,501 CHF hospital admissions for CHF also reported a similar trend in association of short-term exposure to PM_2.5_ and hospitalizations for CHF [11].

Compared to what has been reported by previous studies in developed countries, we found the estimated effects of PM_2.5_ on CHF hospitalizations were less pronounced, but were in line with some relevant studies in China, such as one that investigated the relationship between PM_2.5_ and daily mortality [17]. Possible reasons for differences in the estimated effects include different ambient particulate chemical constitutes [27,28] and their specific sources [29] and the susceptibility of exposed individuals [17]. In the first multicity study to investigate the association of air pollution with CHF morbidity in China, Liu et al. [11] found that patients with preexisting diabetes or hypertension may be more susceptible to PM_2.5_.

In our study, women patients and individuals aged more than 65 years were more susceptible to ambient PM_2.5_. Other studies have also shown that patients at higher risk of admission for CHF are more likely to be older, women, and to have multiple coexisting medical conditions [11]. To evaluate the association between exposure to PM_2.5_ and onset of exacerbation of symptoms leading to hospital admission, Symons et al. [25] assigned their cases into three index times (8-h and 24-h intervals since onset of symptom and date of hospital admission) and found that an IQR increase in PM_2.5_ was associated with hospital admissions for CHF at 2-days lag on value of OR of 1.09 (95% CI: 0.91–1.30%) after controlling for weather conditions among cases defined by 8-h symptom onset. It should also be noted that admission for CHF rather than the timing of onset of symptom was the primary outcome of interest in the study, as it is in many other published studies [11,24,25]. Liu et al. [11] postulated that the delay between the onset of symptoms and admission may be partly attributed to the effects of air pollution having a long latency.

## 5. Limitations

The present study had some potential limitations. First, misclassification of exposure may be unavoidable because the concentrations of PM_2.5_ were collected only from a fixed monitoring station and persons requiring admissions may have had less exposure to PM_2.5_ than healthy individuals because their physical activity was limited. These factors may have resulted in underestimating the effects of air pollution [30]. Second, because we had insufficient data, we were unable to evaluate the independent effects of PM_2.5_ on admission for CHF by using two-pollutant models. Third, as many other studies whose data were from larger admissions databases, the present study also could not take some potential confounders into consideration, such as socioeconomic status, physical activities, and preexisting diseases. Chronic disease burden or lifestyle might also modify the association between PM_2.5_ and heart failure. Further investigation with more detailed information on individuals’ lifestyle risk factors is required to confirm this finding.

## 6. Conclusions

In conclusion, we found significant associations between short-term exposure to PM_2.5_ and hospital admission for CHF in this citywide study. Our findings may contribute to the limited scientific evidence about the acute impacts of PM_2.5_ on CHF in China.

## Figures and Tables

**Table 1:** Characteristics of patients hospitalized for congestive heart failure (CHF).

Characteristics	Hospital Admissions (*n* = 15,256)
Age (year)	
<65 (%)	3210 (21.0%)
≥65 (%)	12,046 (79.0%)
Gender	
Male (%)	8523 (55.9%)
Female (%)	6733 (44.1%)

**Table 2:** Distribution of hospitalization for CHF per day, daily particulate matter (PM_2.5_) concentrations and meteorological conditions.

Variables	Mean ± SD	Minimum	Percentile			Maximum	IQR
			25th	50th	75th		
Daily hospital admissions	16.7 ± 12.1	0	3	18.5	25	59	22
PM_2.5_ (μg/m^3^)	99.5 ± 75.3	7.2	42.5	82.8	133.3	492.8	90.8
Temperature (°C)	12.6 ± 11.6	−12.5	1.5	14.1	23.8	34.5	22.3
Relative humidity (%)	48.6 ± 20.3	9	30	48	66	92	36

**Table 3:** Percentage changes with 95%CI hospitalization for CHF associated with a 10 μg/m^3^ increase in PM_2.5_ for different lag structures.

Lag Days	Percentage Change	95% CI	*p*
Lag 0 days	0.35	0.06–0.64	0.0191
Lag 1 days	0.42	0.17–0.67	0.000899
Lag 2 days	0.31	0.10–0.53	0.00426
Lag 3 days	0.16	−0.06–0.38	0.148
Lag 0–1 day	0.59	0.26–0.91	0.000448
Lag 0–2 days	0.65	0.32–0.99	0.000115
Lag 0–3 days	0.61	0.27–0.95	0.000436

**Table 4:** Percentage changes with 95% CIs in hospitalization for CHF associated with a 10 μg/m^3^ increase in PM_2.5_ by season, sex, and age ^a^.

Variables	Percentage Change	95% CI	*p*
Gender			
Male	0.31	−0.05–0.67	0.09
Female	0.43	−0.005–0.87	0.0527
Age (year)			
<65	0.28	−0.27–0.82	0.318
≥65	0.32	−0.005–0.65	0.0536

^a^ Lag 0 concentrations were used.

**Table 5:** Percentage changes with 95% CIs in hospitalization for CHF associated with a 10 μg/m^3^ increase in PM_2.5._

Variables	df ^a^	Percentage Change	95% CI	*p*
Calendar time	4	0.33	0.07–0.60	0.0142
	5	0.35	0.07–0.62	0.0148
	6	0.33	0.06–0.61	0.0171
	7 ^b^	0.35	0.06–0.64	0.0191
	8	0.40	0.10–0.70	0.00804
Temperature	2	0.35	0.06–0.64	0.0191
	3 ^b^	0.35	0.06–0.64	0.0191
	4	0.47	0.20–0.74	0.000609
	5	0.46	0.19–0.73	0.000874
	6	0.45	0.18–0.72	0.00101
Relative humidity	2	0.35	0.06–0.64	0.0191
	3 ^b^	0.35	0.06–0.64	0.0191
	4	0.33	0.04–0.63	0.0238
	5	0.33	0.04–0.63	0.0239
	6	0.34	0.05–0.63	0.0211

^a^ df notes degree of freedom. ^b^ The df values used in this study model.
